# To the Editors of the Medical and Physical Journal

**Published:** 1801-02

**Authors:** James Woodforde

**Affiliations:** Castle-Cary


### *To the Editors of the Medical and Phyfical Journal.*

Gentlemen,

THE efficacy of Inoculation of the Matter of Cow-pox, in rendering the human body unfufceptible of Small-pox, *appears* now to be fully and completely eftablifhed; at leaft, *as far as we can conclude from the experiments* hitherto made, and the time that has elapfed fince their inftitution.

Every generous mind will congratulate himfelf on a difcovery that promifes fo much benefit to mankind, and will experience the higheft pleafure from the fuccefsful labours of thofe who have fo ardently exerted themfelves in the caufe of humanity and fcience.

I feel myfelf deeply interefted in the fuccefs of this Inoculation; and it is with reluctance I here take the liberty of fubmitting a Cafe, that feems to militate againft the permanent preventive influence of the Variolæ Vaccinæ.

This is the grand defideratum, the determination of which can be effected only by length of time. Three weeks fince, I was requefted to vifit Mrs. Dredge, aged 55 years, living in the neighbourhood of Caftle-Cary, whom I found labouring under Small-pox of the diftinct fort, which fhe caught from a fervant-boy living in her houfe. She informed me, that fhe did not expect this difeafe, fince fhe had taken, twenty-eight years before, the Cow-pox from milking cows affected with the fame. She obferved her difeafe was very fevere, having had numerous puftules on the hands and fingers; loft two nails; had a confiderable tumour in the axilla, and great pyrexia. Sixteen years ago, fhe was much expofed to Small-pox in her own family, having children ill of it, both naturally and from inoculation, and whom fhe conftantly attended, but efcaped infection. She fays, that fhe has not been fubfequently expofed to it till the time abovementioned. Her fifter was affected with Cow-pox at the fame time, and from the fame cows; had a large tumour in the axilla, feveral puftules, and much pyrexia. Twenty years afterwards this woman attended her own children in the natural Small-pox, (one of whom died) but without receiving any infection. The difeafe among the cows extended through the whole dairy, confifting of about twenty cows; and the milk was fo much affected, as to be rendered unfit for ufe for fome time.

The above Hiftory may be confidered as an addition and illuftration of the two Cafes communicated by Dr. Sims, in the firft and fecond volumes of the Medical and Phyfical Journal.

Journal. It will probably be faid, that, from the large and decifive experience of the infallibility of the Cow-pox virus in preventing Small-pox, little credit or confidence can or ought to be given to the few and almoft folitary oppofing inftances now adduced, and efpecially as fome dubiety remains of the genuine nature of the difeafe. I admit the propriety and force of this reafoning, but cannot at the fame time, withhold this information from the public eye. I am, &c.

JAMES WOODFORDE, M. D.

*Castle-Cary, Dec.* 5, 1800.

---

